# Velocity mode transition of dynamic crack propagation in hyperviscoelastic materials: A continuum model study

**DOI:** 10.1038/srep42305

**Published:** 2017-02-10

**Authors:** Atsushi Kubo, Yoshitaka Umeno

**Affiliations:** 1 Institute of Industrial Science, the University of Tokyo, 4-6-1 Komaba, Meguro-ku, Tokyo 153-8505, Japan

## Abstract

Experiments of crack propagation in rubbers have shown that a discontinuous jump of crack propagation velocity can occur as energy release rate increases, which is known as the “mode transition” phenomenon. Although it is believed that the mode transition is strongly related to the mechanical properties, the nature of the mode transition had not been revealed. In this study, dynamic crack propagation on an elastomer was investigated using the finite element method (FEM) with a hyperviscoelastic material model. A series of pure shear test was carried out numerically with FEM simulations and crack velocities were measured under various values of tensile strain. As a result, our FEM simulations successfully reproduced the mode transition. The success of realising the mode transition phenomenon by a simple FEM model, which was achieved for the first time ever, helped to explain that the phenomenon occurs owing to a characteristic non-monotonic temporal development of principal stress near the crack tip.

Rubbers, or elastomers, have exotic mechanical properties, such as entropic elasticity, viscosity and incompressibility. Due to such properties rubbers find various industrial applications, *e.g.*, tyres, dampers, etc. From the viewpoint of industry, it is important to understand the fracture mechanism of rubbers for better design of reliable rubber products. The relationship between the velocity of the crack propagation and the energy release rate has been vigorously investigated as a fundamental property to evaluate how easily rubber products are damaged under external loading. A large number of studies have been carried out to investigate the dynamic crack propagation in rubber materials, including experiments^1^,^2^,^3^,^4^,^5^,^6^, theoretical studies^7^,^8^,^9^,^10^,^11^ and numerical analyses^12^,^13^,^14^,^15^,^16^,^17^,^18^.

Some experiments for the crack propagation in rubbers reported an interesting phenomenon: The crack velocity shows a discontinuous jump as energy release rate increases, which is called a “mode transition”^5^,^6^. There are two mode regions, namely the “slow mode” with low crack velocity at low energy release rates and the “fast mode” with high crack velocity at high energy release rates. The two propagation modes show distinct features such as roughness on the crack surface, which implies highly complicated physics behind the mode transition. The mode transition phenomenon is industrially important because it is empirically known that the property of the mode transition is related to lifetime of rubber products; *i.e.*, the higher the transition energy (energy release rate at the mode transition) becomes, the higher durability the rubber has. There is another kind of transition of the crack propagation in rubber materials that occurs at the vicinity of the sound speed (subsonic-supersonic transition) and has been investigated intensively^16^,^19^,^20^,^21^,^22^. The mechanism of the subsonic-supersonic transition is theoretically well explained^16^,^20^ and this phenomenon has been reproduced successfully by numerical simulations^16^,^22^. On the other hand, the mechanism of the slow-fast mode transition, which is found around the crack velocity much lower than the sound speed in the material, has not been revealed because of the lack of a simple model to reproduce the phenomenon. While Carbone and Persson^11^ have theoretically explained the discontinuity of the mode transition through the effect of the locally high temperature (“hot crack”), their explanation stands only when the crack grows very fast and, in reality, the mode transition can be observed at a low crack velocity. In addition, the hot crack model presupposes a quite high temperature on the crack tip. Thus, there is still room for discussion about the mechanism of the slow-fast mode transition on the dynamic crack propagation in rubbers.

Furthermore, no numerical simulation has successfully explained or reproduced the mode transition phenomenon thus far while some simulations have been performed for the dynamic crack propagation in rubber materials^12^. It is also unknown what relationship exists between the transition energy and the mechanical properties. It is of great importance to reveal the mechanism of the mode transition for industry because it would lead to new designs for rubber materials with improved durability.

In this study, we aim at revealing the nature of the mode transition phenomenon by performing numerical simulation based on the finite element method (FEM). This paper demonstrates that, with proper descriptions of hyperelastic and viscoelastic properties, it is possible to realise the mode transition phenomenon within the framework of FEM. It is observed how the elements on the crack path act during the crack propagation and the viscoelastic behaviours are compared between the slow and fast modes. Then we discuss the relationship between the mode transition phenomenon and the mechanical response at the crack tip.

## Results

### Crack velocity

**Figure 1:**
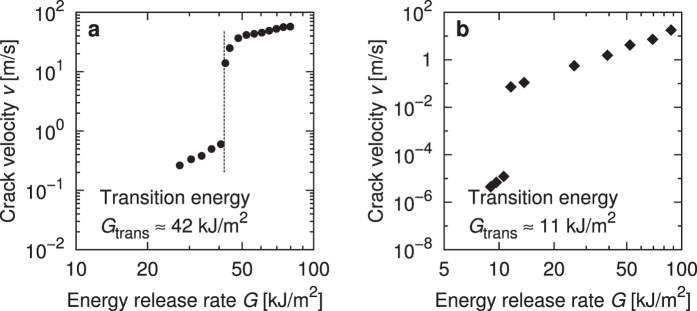
Crack velocity as a function of energy release rate obtained by (**a**) FEM simulation in this study and (**b**) experiment by ref. 6. The transition energy *G*_trans_ indicates the energy release rate at the mode transition. Note that the figures are plotted on a different scale.

Figure 1(a) shows the relationship between the crack propagation velocity *ν* and the energy release rate *G* obtained by the FEM simulation, compared with an experiment^6^ (Fig. 1(b)). It was observed that the crack velocity changes discontinuously around the energy release rate of G ≈ 42 kJ/m^2^, which is referred to as the transition energy *G*_trans_. This jump corresponds to the slow-fast mode transition observed in experiments^5^,^6^. The crack velocity changes at the transition by nearly 2 orders of magnitude in this simulation. To the best of our knowledge, this is the first FEM result ever that has succeeded in explicitly reproducing the mode transition in the crack propagation.

On the whole, the simulation result is not in a quantitative agreement with the corresponding experiment. The crack velocity and the transition energy are overestimated in the simulation, *e.g. G*_trans_ ≈ 11 kJ/m^2^ in the experiment^6^. Note that our FEM results inevitably include a quantitative deviation from experiments because of the finiteness of elements, especially in the vicinity of the crack tip. Moreover, the criterion of fracture, which we assume is stipulated by the critical principal stress, may be too simple to achieve a quantitative agreement with the experiments. In this study, we basically focus on the qualitative aspect of the simulation results because, as is explained in the Discussion section, the qualitative aspect is helpful enough to understand the basic mechanism of the mode transition.

### Crack profile

It was observed that the crack propagated along a straight path in the *x*-axis without any oscillation or roughness regardless of the mode type while an experiment reported the formation of rough crack surfaces in the slow mode and smooth crack surfaces in the fast mode^5^. This disagreement is basically likely to be attributed to the finiteness of the elements (aligned along the crack path) and the size of elements, which is not fine enough to describe rough crack surfaces.

**Figure 2:**
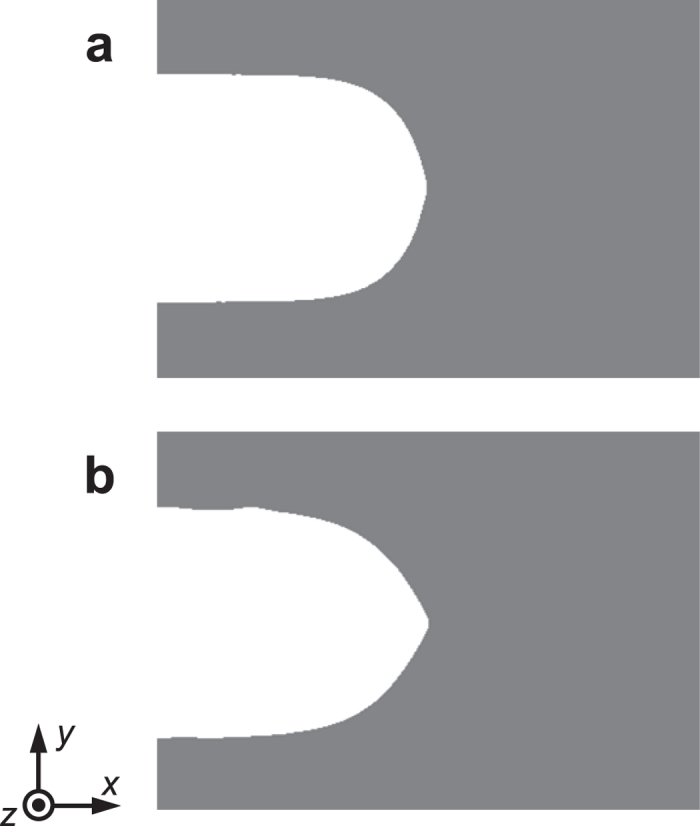
Shape of propagating crack tip at two different energy release rates, *G*. (**a**) *G* = 27 kJ/m^2^ and (**b**) *G* = 56 kJ/m^2^.

The shape of the crack tip was observed to become sharper as a larger strain is applied. Figure 2 shows the shapes of the crack tip under two different energy release rates, *i.e.*, different applied strains *ε*_load_. While the crack shape is blunt at the lower energy release rate (Fig. 2(a)), the shape becomes sharp at the higher energy release rate (Fig. 2(b)). This result is in a qualitative agreement with experiments^6^. It may be worth noting that the change of the crack shape is not discontinuous at the mode transition point in contrast to the clear discontinuity in the crack velocity.

In the slow mode, it was observed that the crack propagates repeating the cycle of two states, namely the state with considerable energy dissipation right after element deletion and the state of a blunt crack after energy dissipation. This observation indicates that fracture in the slow mode occurs basically after energy dissipation nearly vanishes. In other words, elasticity mainly contributes to stress on the crack-tip elements at the moment of fracture rather than viscosity does. On the other hand, in the fast mode, crack propagates steadily, which suggests that both elasticity and viscosity contribute to stress on the crack-tip element at the moment of fracture.

### Mechanical response of crack-tip element

**Figure 3:**
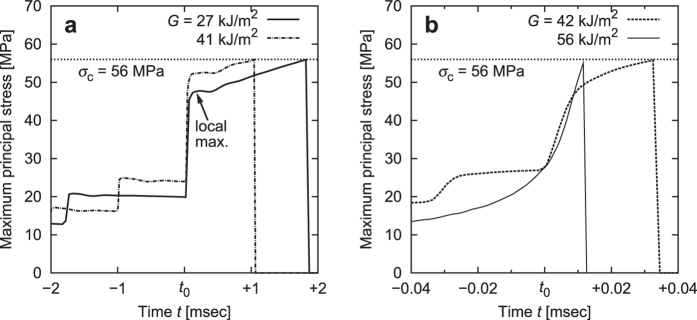
Maximum principal stress on an element on the crack path as a function of time. (**a**) Slow mode (*G* = 27 and 41 kJ/m^2^). (**b**) Fast mode (*G* = 42 and 56 kJ/m^2^). The element faces the carck tip at time *t* = *t*_0_. Note that the magnitude of absissae in figures are different by about 2 orders.

Figures 3(a,b) show the maximum principal stress exerted on the elements on the crack path in the slow and fast modes, respectively. In both modes, the principal stress on the element increases suddenly when the element faces the crack tip (shown as *t* = *t*_0_ in Fig. 3). After a certain time, the principal stress reaches the critical stress *σ*_c_ and then drops instantly to naught because the element is deleted. It is important to note the following characteristic difference between the two modes: In the slow mode, stress on a crack-tip element rapidly increases and reaches a *local maximum point*. Then the stress exhibits a slight decrease and turns to increase again until fracture. In the fast mode, in contrast, the stress at the crack tip exhibits a *monotonic increase* until fracture. Moreover, the two cases of the slow mode presented in Fig. 3(a) exhibit temporal development of the stress similar to each other. The difference in the mechanical response around the crack tip plays an essential role in the mode transition.

## Discussion

**Figure 4:**
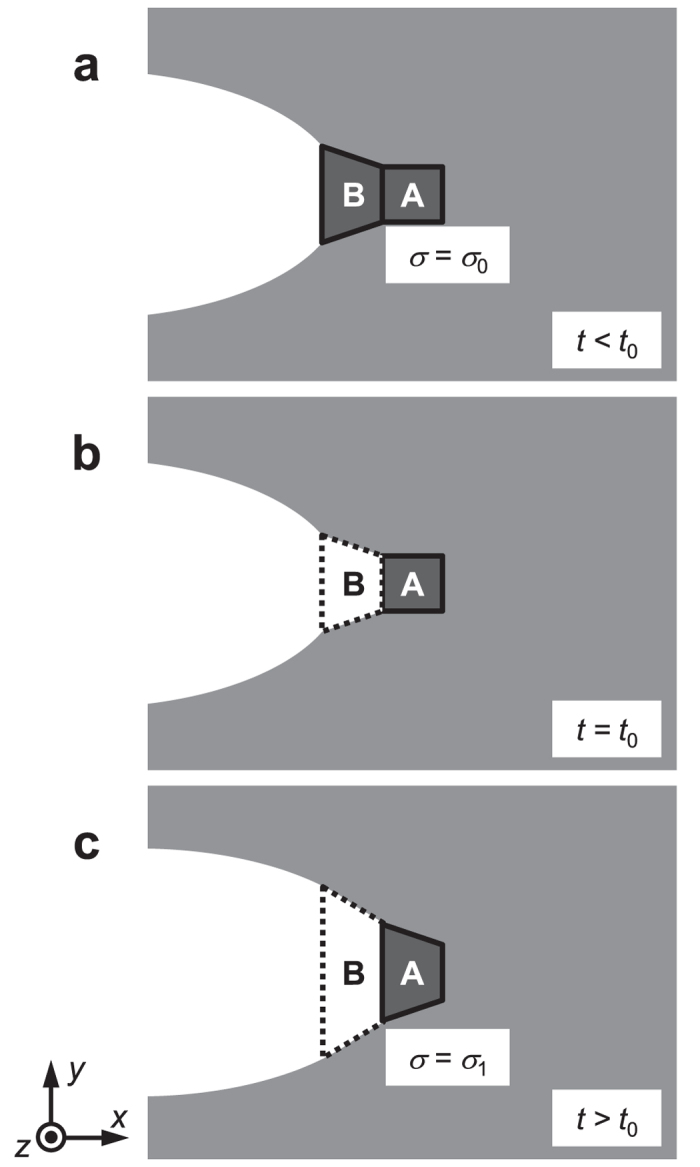
Schematic illustration of elements in the vicinity of crack tip. (**a**) The element A, next to the crack, holds stress *σ* = *σ*_0_ at *t* = *t*_0_. (**b**) The crack tip element B is deleted at *t* = *t*_0_. (**c**) At *t* > *t*_0_, the structural relaxation proceeds to the new equilibrium state, where the element A holds *σ* = *σ*_1_.

Let us consider the mechanical response of the element at the crack tip to understand the mechanism of the mode transition in this simulation. Here, we suppose the following process:

1. Two elements, A and B, on the crack path are considered. The element B locates on the crack tip and the element A sites next to the element B at the initial state (Fig. 4(a)). The elements A and B have been fully relaxed at time *t* < *t*_0_. The element A stores stress *σ* = *σ*_0_ at this state.
2. The element B is broken (deleted) at time *t *= *t*_0_ (Fig. 4(b)). The structure has not yet been relaxed at this moment.
3. The element A, which newly faces the crack tip, gets relaxed to the new equilibrium state with stress *σ* = *σ*_1_ (Fig. 4(c)).

Furthermore, the stress and strain are regarded as scalar values for simplification, which should be reasonable since the load is uniaxial. As shown in Fig. 3(a), the element A is expected to undergo a local maximum of *σ(t*) at *t* > *t*_0_.

**Figure 5:**
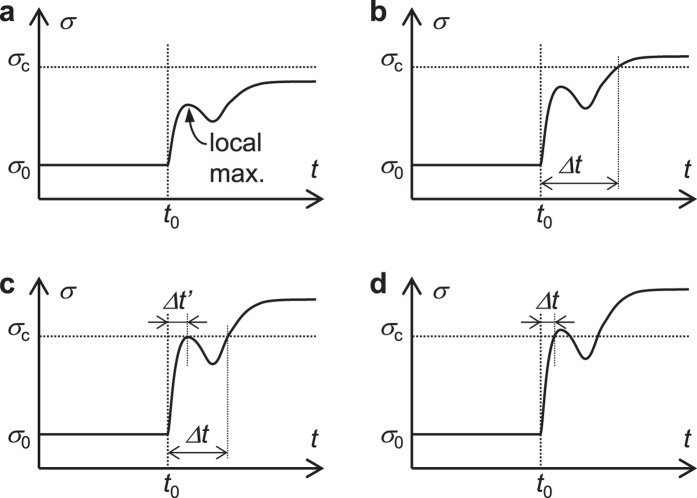
Schematic illustration of the mechanical response of the element on the crack tip. The characteristics of the curve shape are exaggerated for clarity. (**a**) No fracture occurs under very low tensile load *ε*_load_. (**b**) Fracture occurs after undergoing local maximum point (slow mode). (**c**) Local maximum in the stress curve reaches the critical stress and the mode transition occurs. (**d**) Fracture occurs within local maximum point (fast mode). Fracture time per element is shown as Δ*t* and Δ*t*′.

If *σ* does not reach the fracture criterion (*σ*_1_ < *σ*_c_), no more crack propagation occurs (Fig. 5(a)). As increasing strain *ε*_load_, *σ* reaches *σ*_c_ after a certain time Δ*t* passes (Fig. 5(b)); *i.e.*, Δ*t* gives the time necessary for fracture of one element. Then the crack velocity is obtained as *l*_*e*_/Δ*t*, where *l*_e_ denotes the element length along the crack path. Under a certain value of strain, the local maximum reaches *σ*_c_, where the time necessary for fracture jumps from Δ*t* to Δ*t*′ (Fig. 5(c)). Around this transition point, both Δ*t* and Δ*t*′ are theoretically possible and either of them actually (in reality) occurs according to various factors of perturbation such as inhomogeneity of a specimen. Thus, the crack propagation mode can be easily switched from the slow mode to the fast mode and *vice versa*. This is presumably the reason why the stick-slip behaviour is observed in the experiments. Indeed, an experiment provided an evidence to regard the stick-slip behaviour as a continual alternation between the slow and fast modes^5^. Under higher strains, the fracture is expected to occur at the local maximum, as shown in Fig. 5(d), leading to the fast mode crack propagation. Interestingly, the basic mechanical response at the crack tip is likely to be common to both the modes; the difference between them is only whether fracture occurs after or before reaching the local maximum in *σ(t*), shown in Fig. 5.

The discussion above is basically based on simple hypotheses such as hyperelasticity, linear viscosity and a fracture criterion based on the principal stress. Thus, those factors are regarded as the sufficient condition for the mode transition phenomenon to occur. The probable least condition required is the distribution of the relaxation time in the representation of viscosity. If there exists a unique relaxation time that dominates all the relaxation behaviour of stress and strain, then the local maximum point is unlikely to appear.

It is found that the observed transition phenomenon is distinguished from the subsonic-supersonic transition through some striking differences between the phenomena: The observed transition is characterised by the discontinuous change of the crack velocity as explained above. Furthermore, in the present analysis the crack velocity at the transition point is sufficiently slow compared with the sound speed. For example, the lowest crack velocity at the fast mode measures 14 m/s (Fig. 1(a)), while the sound speed *c*_s_ of rubber materials is typically about 50 m/s (according to the evaluation from the first-order shear modulus *μ*_(1)_ and mass density *ρ*, 
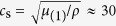
 m/s for the compound under consideration^6^). In contrast to our FEM simulation results, the subsonic-supersonic transition is a continuous change of the crack velocity at the vicinity of the sound speed, as shown in experiments and numerical analyses (see *e.g.* Fig. 3 in ref. 22).

There are some non-trivial factors that were omitted in the discussion above; *e.g*., energy dissipation at the process zone, inertia effect, finite deformation, etc., which are implicitly included in the FEM simulation. Obviously, the factors can quantitatively contribute to the crack propagation behaviour. Nevertheless, they can be regarded as marginal factors when we focus on the essential mechanisms of the mode transition. It is important to note that the mode transition can be successfully explained through the characteristic mechanical response on the crack-tip element despite the omission of those factors. Besides the factors above, the present FEM simulations do not treat other possible factors, such as temperature distribution, plastic deformation and the Mullins effect^23^,^24^. It will be necessary to include those effects in order to perform more realistic simulations.

While the mode transition phenomenon was clearly explained through the characteristic mechanical response at the crack tip, it is necessary to thoroughly reveal the origin of such mechanical response. In addition, there is room for investigation in quantitative aspect of the mode transition, *e.g.*, evaluation of the transition energy. These subjects will be our future work.

## Method

### Constitutive model

The constitutive model of rubber material in this study consists of hyperelasticity and linear viscosity. The hyperelastic and viscous terms represent the static and rate-dependent responses, respectively. The total stress tensor is given by the sum of both contributions (referred to below as 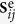 and 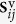). Hyperelasticity independent of rate effect is described by the Ogden model^25^. The Ogden model gives the strain energy density *W* as follows:


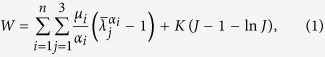


where *K* and *J* denote the bulk modulus and the relative volume, respectively. The bulk modulus *K* was determined through the Poisson’s ratio, which was set to 0.495 in this study. The series of 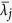 denotes the principal stretch divided by *J*. Material parameters *μ*_*i*_ and α_*i*_ are empirically determined. In this study, the number of terms for the Ogden model, *n*, was set to 3. The strain density function gives the second Piola-Kirchhoff stress tensor of hyperelasticity, 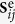, as follows:


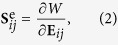


where **E**_*ij*_ denotes the Green-St. Venant strain tensor. The effect of viscosity on stress, 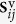, is given by the following convolution integral;


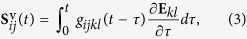


where *g(t*) denotes the relaxation function. The Einstein’s summation convention is applied for indices *k* and *l*. We adopted the Prony series for *g(t*), defined as


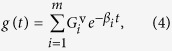


where series of 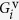, and *β*_*i*_ are material parameters. The number of terms for the Prony series, *m*, was set to 12, in this study. The Prony series description is equivalent to the Maxwell model.

The material parameters in the constitutive equations were determined in such a way that they reproduce the uniaxial stress-strain curve at a low strain rate and the stress relaxation curve obtained by experiments for a cross-linked acrylonitrile-butadiene rubber (35% acrylonitrile content) with carbon black filler (0.05 volume fraction) shown in ref. 6. The determined material parameters are listed in Supplementary Table S1. It was confirmed that both the uniaxial stress-strain relationship and the stress relaxation function were reproduced well by the constitutive model in the wide range of strain and time (see Supplementary Fig. S1).

The fracture criterion was given by the maximum principal stress. If an element holds a principal stress as high as the given critical stress, *σ*_c_, then the element is deleted to realise fracture. The critical principal stress was estimated through the experimental result of uniaxial tensile strength under the quasi-static condition. Since the experimental strength was measured as the nominal stress, it was converted into the true stress on the supposition that the volume is constant under the quasi-static uniaxial tension.

In our model, the Mullins effect^23^,^24^, which is known as the hysteresis effect of mechanical properties observed in rubber materials, was not considered for simplification.

### Simulation setup

We carried out FEM simulations of dynamic crack propagation mimicking the pure shear test, where the crack is expected to propagate at a constant velocity under a given strain. We adopted the FEM simulation package LS-DYNA (Livermore Software Technology Corporation).

According to Rivlin and Thomas^1^,^26^, the energy release rate in the pure shear test, *G*, is given as the product of the strain energy density *W* and the width of the unstrained specimen, *h*_0_;


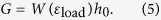


Equation 5 indicates that *G* is simply given as a function of applied strain, *ε*_load_. Here, *W* is calculated with the Ogden model (1) under the pure shear condition; *i.e.*, 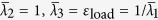.

The length (in the *x*-axis), the width (in the *y*-axis) and the thickness (in the *z*-axis) of the specimen were set to 180 mm, 20 mm and 1 mm, respectively. The dimension of the elements on which the crack was expected to propagate was set to 0.50 mm, 0.19 mm and 0.20 mm in the *x*-, *y*- and *z*-axes, respectively. As explained in the previous section, the elements were to be deleted when the maximum principal stress reaches the critical stress, *σ*_c_ = 56 MPa, to describe the crack propagation.

**Figure 6:**
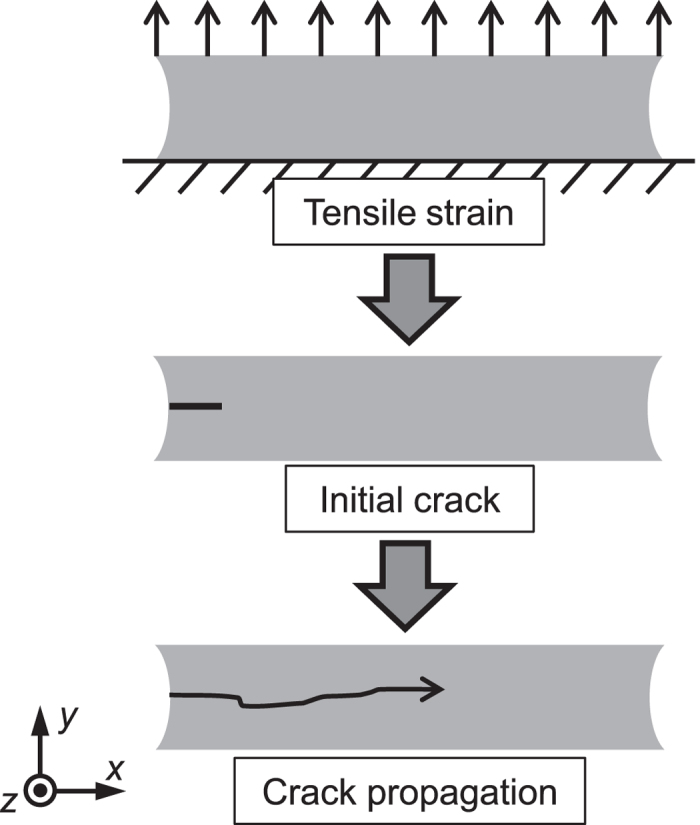
Schematic illustration of simulation procedure. A specimen is set thin along the *z*-axis to realise the pure shear condition. Firstly, a specimen is stretched along the *y*-axis to a predetermined strain. Secondly, initial crack is introduced by removing elements. Then crack starts propagating along the *x*-axis and the velocity of crack propagation is measured. The relationship between the crack velocity and the applied strain by repeating this procedure with various strains.

Figure 6 schematically shows the procedure of simulation. Tensile strain *ε*_load_ was loaded in the *y*-direction by controlling the displacement on the top *xz*-plane at a constant strain rate 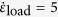 s^−1^. The degrees of freedom regarding the displacement along all the directions on the bottom *xz*-plane and those along the *x*- and *z*-directions on the top *xz*-plane were fixed during the whole simulation. The degree of freedom along the *y*-direction on the top plane was also fixed after the tensile strain control.

Right after reaching the predetermined strain, the initial crack was introduced with a length of 10 mm in the *x*-direction by removing elements. When the crack started propagating spontaneously and steadily, the rate of propagation (crack velocity) was measured. Since the energy release rate *G* can be calculated with the applied strain *ε*_load_, we obtained the relationship between the energy release rate and the crack velocity.

## Additional Information

**How to cite this article:** Kubo, A. and Umeno, Y. Velocity mode transition of dynamic crack propagation in hyperviscoelastic materials: A continuum model study. *Sci. Rep.* **7**, 42305; doi: 10.1038/srep42305 (2017).

**Publisher's note:** Springer Nature remains neutral with regard to jurisdictional claims in published maps and institutional affiliations.

## Supplementary Material

Supplementary Information

## References

[b1] Rivlin R. S. & Thomas A. G. Rupture of rubber. i. characteristic energy for tearing. J. Polym. Sci. 10, 291–318 (1953).

[b2] Mullins L. Rupture of rubber, part ix: role of hysteresis in the tearing of rubber. Trans. Inst. Rubber Ind. 35, 213–222 (1959).

[b3] Gent A. N. & Marteny P. Crack velocities in natural rubber. J. Mater. Sci. 17, 2955–2960 (1982).

[b4] Gent A. N. Adhesion and strength of viscoelastic solids. is there a relationship between adhesion and bulk properties? Langmuir 12, 4492–4496 (1996).

[b5] Tsunoda K., Busfield J., Davies C. & Thomas A. Effect of materials variables on the tear behaviour of a non-crystallising elastomer. J. Mater. Sci. 35, 5187–5198 (2000).

[b6] Morishita Y., Tsunoda K. & Urayama K. Velocity transition in the crack growth dynamics of filled elastomers: contributions of nonlinear viscoelasticity. Phys. Rev. E 93, 043001 (2016). 27176379 10.1103/PhysRevE.93.043001

[b7] Schapery R. A. A theory of crack initiation and growth in viscoelastic media. Int. J. Fract. 11, 141–159 (1975).

[b8] Barber M., Donley J. & Langer J. S. Steady-state propagation of a crack in a viscoelastic strip. Phys. Rev. A 40, 366–376 (1989). 10.1103/physreva.40.366 9901903

[b9] Greenwood J. A. The theory of viscoelastic crack propagation and healing. J. Phys. D: Appl. Phys. 37, 2557–2569 (2004).

[b10] Persson B. N. J. & Brener E. A. Crack propagation in viscoelastic solids. Phys. Rev. E 71, 036123 (2005). 10.1103/PhysRevE.71.036123 15903509

[b11] Carbone G. & Persson B. N. J. Crack motion in viscoelastic solids: the role of the flash temperature. Eur. Phys. J. E 17, 261–281 (2005). 15997339 10.1140/epje/i2005-10013-y

[b12] Kroon M. Steady-state crack growth in rubber-like solids. Int. J. Fract. 169, 49–60 (2011).

[b13] Elmukashfi E. & Kroon M. Numerical analysis of dynamic crack propagation in rubber. Int. J. Fract. 177, 163–178 (2012).

[b14] Kroon M. Energy release rates in rubber during dynamic crack propagation. Int. J. Solids Struct. 51, 4419–4426 (2014).

[b15] Elmukashfi E. & Kroon M. Numerical analysis of dynamic crack propagation in biaxially strained rubber sheets. Eng. Fract. Mech. 124, 1–17 (2014).

[b16] Marder M. Shock-wave theory for rupture of rubber. Phys. Rev. Lett. 94, 048001 (2005). 15783601 10.1103/PhysRevLett.94.048001

[b17] Wang W. & Chen S. Hyperelasticity, viscoelasticity, and nonlocal elasticity govern dynamic fracture in rubber. Phys. Rev. Lett. 95, 144301 (2005). 16241660 10.1103/PhysRevLett.95.144301

[b18] Horst T. & Heinrich G. Crack propagation behavior in rubber materials. Polym. Sci. Ser. A 50, 583–590 (2008).

[b19] Petersan P. J., Deegan R. D., Marder M. & Swinney H. L. Cracks in rubber under tension exceed the shear wave speed. Phys. Rev. Lett. 93, 015504 (2004).

[b20] Marder M. Supersonic rupture of rubber. J. Mech. Phys. Solids 54, 491–532 (2006).

[b21] Zhang H. P., Niemczura J., Dennis G., Ravi-Chandar K. & Marder M. Toughening effect of strain-induced crystallites in natural rubber. Phys. Rev. Lett. 102, 245503 (2009). 19659026 10.1103/PhysRevLett.102.245503

[b22] Chen C. H., Zhang H. P., Niemczura J., Ravi-Chandar K. & Marder M. Scaling of crack propagation in rubber sheets. Europhys. Lett. 96, 36009 (2011).

[b23] Mullins L. Effect of stretching on the properties of rubber. Rubber Chem. Technol. 21, 281–300 (1948).

[b24] Mullins L. Thixotropic behavior of carbon black in rubber. Rubber Chem. Technol. 23, 733–743 (1950).

[b25] Ogden R. Large deformation isotropic elasticity – on the correlation of theory and experiment for incompressible rubberlike solids. Proc. R. Soc. A 326, 565–584 (1972).

[b26] Thomas A. G. Rupture of rubber. ii. the strain concentration at an incision. J. Polym. Sci. 18, 177–188 (1955).

