# Supplementary material for: Velocity mode transition of dynamic crack propagation in hyperviscoelastic materials: A continuum model study
Source: Sci Rep. 2017 Feb 10;7:42305. doi: 10.1038/srep42305 (PMC5301218; doi:10.1038/srep42305)
Supplement: Supplementary Information [file srep42305-s1.pdf]

Supplementary Information

Velocity mode transition of dynamic crack propagation in hyperviscoelastic materials: A continuum model study

Atsushi Kubo[1,*] **and Yoshitaka Umeno**[1]

[1]Institute of Industrial Science, the University of Tokyo, 4-6-1 Komaba, Meguro-ku, Tokyo 153-8505, Japan
[*]kubo@ulab.iis.u-tokyo.ac.jp

References

1. Morishita, Y., Tsunoda, K. & Urayama, K. Velocity transition in the crack growth dynamics of filled elastomers: contributions of nonlinear viscoelasticity. *Phys. Rev. E* **93**, 043001 (2016).

Supplementary Table S1. Material parameters for Ogden model and Prony series. The first parameter pair of the Prony series, G_1^{v} and β_1, was ignored in the actual simulations because that represents the static behaviour, which is already considered in the Ogden model.

i	μ_i [MPa]	α_i	G_i^{v} [MPa]	β_i [msec^{-1}]
1	0.0511	3.96	0.839	0
2	−0.105	2.78	0.404	10^{-5}
3	9.44	0.206	0.00	10^{-4}
4			0.464	10^{-3}
5			0.375	10^{-2}
6			0.00	10^{-1}
7			1.98	1
8			0.00	10
9			4.31	10^2
10			58.4	10^3
11			77.1	10^4
12			405	10^5

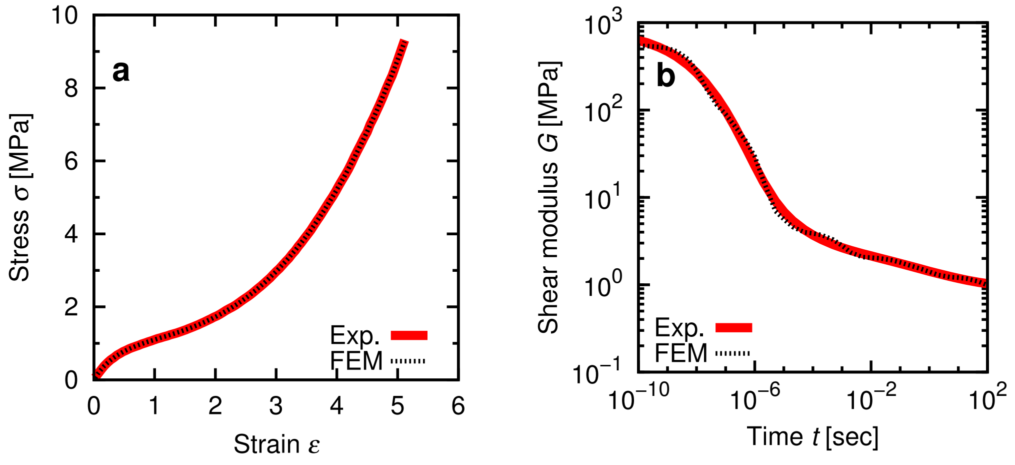

Supplementary Figure S1. (a) Nominal stress-strain relationship under quasi-static uniaxial tension. (b) Shear stress relaxation curve. The experimental results are from Morishita *et al.*[1]
